# An imaging-based RNA interference screen for modulators of the Rab6-mediated Golgi-to-ER pathway in mammalian cells

**DOI:** 10.3389/fcell.2022.1050190

**Published:** 2022-11-29

**Authors:** Linda F. Heffernan, Pia M. Suckrau, Teerna Banerjee, Margaritha M. Mysior, Jeremy C. Simpson

**Affiliations:** Cell Screening Laboratory, UCD School of Biology and Environmental Science, University College Dublin, Dublin, Ireland

**Keywords:** membrane trafficking, Rab6-dependent retrograde pathway, Rab GTPases, SNARE, SM, SCFD1/SLY1

## Abstract

In mammalian cells, membrane traffic pathways play a critical role in connecting the various compartments of the endomembrane system. Each of these pathways is highly regulated, requiring specific machinery to ensure their fidelity. In the early secretory pathway, transport between the endoplasmic reticulum (ER) and Golgi apparatus is largely regulated *via* cytoplasmic coat protein complexes that play a role in identifying cargo and forming the transport carriers. The secretory pathway is counterbalanced by the retrograde pathway, which is essential for the recycling of molecules from the Golgi back to the ER. It is believed that there are at least two mechanisms to achieve this - one using the cytoplasmic COPI coat complex, and another, poorly characterised pathway, regulated by the small GTPase Rab6. In this work, we describe a systematic RNA interference screen targeting proteins associated with membrane fusion, in order to identify the machinery responsible for the fusion of Golgi-derived Rab6 carriers at the ER. We not only assess the delivery of Rab6 to the ER, but also one of its cargo molecules, the Shiga-like toxin B-chain. These screens reveal that three proteins, VAMP4, STX5, and SCFD1/SLY1, are all important for the fusion of Rab6 carriers at the ER. Live cell imaging experiments also show that the depletion of SCFD1/SLY1 prevents the membrane fusion event, suggesting that this molecule is an essential regulator of this pathway.

## 1 Introduction

Compartmentalisation allows mammalian cells to execute specific biochemical reactions in a spatially and temporally separated manner. The compartments of the endomembrane system are connected by various pathways that allow the highly regulated transport of cargo from one compartment to another (Behnia and Munro, 2005). The early anterograde pathway between the endoplasmic reticulum (ER) and Golgi apparatus is well characterised and is dependent on coat protein complex II (COPII). The COPII coat plays a role in cargo identification and physical reshaping of the ER membrane to form carriers that facilitate the transport of newly synthesised cargo from the ER to the Golgi complex (Peotter et al., 2019). This pathway is counterbalanced by the Golgi-to-ER retrograde pathway that is dependent on coat protein complex I (COPI). The COPI-dependent pathway is essential for the retrieval of machinery molecules involved in the anterograde pathway, unfolded proteins that may have escaped the ER, and the recycling of resident ER proteins (Arakel and Schwappach, 2018). The structures of the COPII and COPI coats are evolutionarily conserved (Lee and Goldberg, 2010). They drive transport vesicle formation using a mechanism initiated through membrane association of a small GTPase. The recruitment of the GTPase is regulated by a guanine nucleotide exchange factor (GEF), which provides a steady supply of the GTP-loaded GTPase on the membrane, allowing the coat to assemble. The further addition of coat proteins induces ever increasing curvature on the membrane until the transport vesicle is released. During the transport of the vesicle to its destination, the coat complex disassembles from the membrane, aided by the activity of a GTPase activating protein (GAP).

On arrival at its target membrane, the vesicle needs to fuse in order to deliver its contents, and this process also requires precise regulation. The two main classes of molecules involved in this step are tethers and SNARE (soluble N-ethylmaleimide sensitive factor attachment protein receptor) proteins. Tethers assist in the initial connection between vesicle and acceptor membrane, bringing them closer together prior to fusion. Broadly, tethers can be classified into two subgroups, namely large homodimeric coiled-coil proteins and multisubunit tethering complexes (Yu and Hughson, 2010). The SNARE and SNARE-related proteins are essential for the vesicle-target membrane fusion event, and they work by building a so-called SNARE complex between cognate SNAREs on the vesicle membrane and acceptor membrane. 47 mammalian members have been identified and each is localised to distinct membranes, thereby playing a key role in determining membrane identity and specificity for fusion. The steps that constitute the fusion events, including SNARE complex pairing, assembly and disassembly, are regulated by Sec1/Munc18 (SM) proteins, tethers, the N-ethylmaleimide-sensitive factor (NSF) and ⍺-SNAP (Wang et al., 2017).

Although the roles of COPII and COPI in regulating membrane dynamics in the early secretory pathway are well-established, an additional Golgi-to-ER transport mechanism is also known to exist. This pathway was initially identified through the observation that exogenously added *E. coli* Shiga-like toxin was still delivered to the ER in the presence of various inhibitors of COPI function. This pathway was found to be dependent on the small GTPase Rab6 and was termed the Rab6-dependent or COPI-independent retrograde pathway (Girod et al., 1999; White et al., 1999). This pathway was also visually distinctive through its use of tubular rather than vesicular transport carriers. Despite the known existence of this pathway for more than 20 years, comparatively little molecular detail of its regulation is understood (Heffernan and Simpson, 2014). It has been suggested that microtubules are necessary for Rab6-dependent transport, however, only a few molecules associated with the microtubule machinery have been identified. Rab6 interacts with the adaptor protein Bicaudal-D (BICD), which in turn recruits and interacts with the dynein-dynactin motor complex (Matanis et al., 2002). Rab6 also interacts directly with the dynactin subunit p150^Glued^ (Short et al., 2002) and with the dynein light chain protein DYNLRB1 (Wanschers et al., 2008). However, further mechanistic studies of the role of microtubules in the Rab6-dependent pathway are needed to understand their involvement fully. This is relevant because the highly pleiomorphic and dynamic nature of Rab6 carriers strongly indicates that active microtubule-mediated mechanisms directly drive their motility.

It remains unclear as to which other factors determine whether cargo is transported *via* vesicles or tubules. Indeed, not only is the type of cargo important, but also its concentration, and evidence exists that tubular carriers in the early secretory pathway have a higher capacity to transport cargo (Simpson et al., 2006). Another potential factor is the local lipid composition at the donor membrane, as it has been shown that some enzymes playing a role in lipid metabolism have an important role in the decision of whether a COPI vesicle carrier for retrograde transport or a COPI tubular carrier for intra-Golgi transport is formed (Yang et al., 2011).

In addition to the question of what cargo is transported *via* the Rab6-dependent retrograde pathway and which factors determine the use of this pathway, other mechanistic details remain to be resolved. Nothing is known about the last step of membrane trafficking events for the Rab6-dependent retrograde pathway, namely the fusion events that take place at the ER. The aim of this study was to screen the SNARE and SNARE-related proteins for a potential role in Rab6 tubule fusion events at the ER, thereby providing the first systematic assessment of the likely molecular machinery involved in this key transport step.

## 2 Materials and methods

### 2.1 Cell culture

HeLa Kyoto cells (human cervical cancer cell line, RRID; CVCL_1922) were routinely cultured at 37°C in Dulbecco’s modified Eagle medium (DMEM) (Life Technologies, Carlsbad, CA, United States) with 1 g/L D-glucose supplemented with 10% foetal calf serum (FCS) (Life Technologies) and 1% L-Glutamine (Life Technologies) in 10 cm Nunclon™ culture dishes. Cells were maintained in a humidified atmosphere of 5% CO_2_/95% air. Upon reaching 80% confluency, cells were subcultured in a 1:10 dilution; cell detachment was facilitated by incubation with 0.05% trypsin-EDTA solution (Life Technologies). Cells were used from passages 1 to 10; following this they were discarded.

### 2.2 DNA plasmids

An mCherry-Rab6A′ expression plasmid was made by subcloning the Rab6A′ open reading frame from a GFP-Rab6A′ expression plasmid into a pmCherry-C1 (Takara Bio, Kusatsu City, Japan) using *Sac*I (New England Biolabs, Ipswich, MA, United States) and *Sac*II (New England Biolabs) restriction sites. The following expression plasmids were used: EGFP-Rab6A, EGFP-Rab6A′, mCh-Rab6A’, and EGFP-KDELR.

### 2.3 DNA and siRNA transfections

One day prior to transfection, cells were seeded into appropriate plates or live-cell imaging dishes. Cells were either transfected with Silencer Select small interfering RNAs (siRNAs, Ambion, Thermo Fisher Scientific, Waltham, MA, United States) or DNA plasmids. Oligofectamine transfection reagent (Life Technologies) was used to transfect cells with siRNAs for 48 h following the manufacturer’s instructions. A list of all used siRNA sequences can be found in Supplementary Table S1. Transit-LT1 transfection reagent (Mirus Bio, Madison, WI, United States) was used to transfect cells with DNA plasmids for up to 24 h or as indicated following the manufacturer’s instructions.

### 2.4 Golgi redistribution assay

Cells were transfected with siRNAs and DNA plasmids according to Section 2.3. Cells were treated with 10 μg/ml Brefeldin A (BFA) (Life Technologies) in DMEM at 37 °C for 15 min. After this, cells were fixed for 20 min with 3% PFA (Sigma, St. Louis, MO, United States) in PBS (Sigma), followed by 5 min quenching with 30 mM glycine (Fisher Scientific, Hampton, NH, United States) in PBS and three PBS washes. Nuclei were stained with 0.2 μg/ml Hoechst 33342 (Sigma) diluted in PBS for 5 min, followed by three PBS washes. If cells were grown on glass coverslips, cells were mounted with Mowiol (Sigma) on glass slides. Images were acquired with an Olympus FluoView FV1000 microscope with a 60x UPLSAPO 1.35 NA oil immersion objective. Two independent biological replicates were performed.

### 2.5 Shiga-like toxin-1B assay

Cells grown in a 24-well plate with a glass coverslip per well were transfected with siRNAs prior to the assay. Cells grown on coverslips were washed with ice-cold PBS and incubated on ice with 1.5 μg/ml Shiga-like toxin-1 B subunit (SLTxB) labelled with Cy3 in PBS (Sigma) for 30 min. After incubation, cells were washed with ice-cold PBS and placed into DMEM supplemented with 10% FBS at 37°C for 4 h. Following the incubation, cells were fixed for 20 min with 3% PFA in PBS, followed by 5 min quenching with 30 mM glycine and three PBS washes. Nuclei were stained with 0.2 μg/ml Hoechst 33342 diluted in PBS for 5 min. Followed by three PBS washes. If cells were grown on glass coverslips, cells were mounted with Mowiol on glass slides. Images were acquired on a Leica DMI6000B inverted wide-field microscope controlled by Leica Application Suite software. A 40x 1.25 NA oil immersion objective was used. Three independent biological replicates were performed.

### 2.6 Live cell imaging

Cells grown in live-cell 35 mm dishes (MatTek, Ashland, MA, United States) were transfected with siRNAs and DNA plasmids. After 19 h of DNA plasmid transfection, cells were treated with 10 μg/ml BFA in FluoroBrite™ DMEM supplemented with 30 mM HEPES (pH 7.5, Sigma). Live cell imaging was performed on an Olympus FluoView FV3000 microscope with a 60x PlanApo 1.4 NA oil immersion objective at 37°C. Time-lapse images were taken every 10 s for a total time of 15 min or until the redistribution of the Golgi marker to the ER after BFA treatment was complete. The time between the addition of BFA and imaging start was stopped and taken into account for analysis. A minimum of 10 cells per condition were analysed.

### 2.7 RNA extraction, cDNA synthesis and real-time quantitative PCR

Cells grown in 12-well plates were transfected with siRNAs for 48 h. Total RNA was extracted using an Invisorb Spin Cell RNA mini kit (Invitek Molecular, Berlin, Germany) according to the manufacturer’s protocol, and RNA concentration was determined using a NanoDrop3000 (Thermo Scientific). 500 ng of total RNA was used to perform cDNA synthesis using a High Capacity cDNA Reverse Transcription kit (Life Technologies) according to the manufacturer’s protocol. Real-time quantitative PCR was performed using Fast SYBR Green PCR MasterMix (Life Technologies) in a 7500 Fast real-time PCR machine (Life Technologies). One-twentieth of the cDNA was used as a template for the qPCR reaction, and 200 nM of each primer was used (see Supplementary Table S2). Three biological replicates were performed. The results were acquired using the -ΔCt method, with mRNA levels from siRNA-treated cells being normalised to those found in cells treated with non-silencing (NEG) siRNAs.

### 2.8 Analysis of tubules

In order to measure membrane tubule length in cells after BFA treatment and the time until Golgi blinkout, ImageJ software was used. The median and maximum intensities within the cell were used to determine the time until blinkout after BFA treatment. For this, a set of time-lapse images were opened in ImageJ, and the brightness and contrast were adjusted to a consistent value before the background was subtracted, utilising the rolling ball algorithm with a rolling ball radius of 50 pixels. Every single cell was manually selected as the region of interest, and the intensity was measured for each timepoint within the selected region. Tubule lengths were measured using the freehand line tool. The maximum length of individual tubules was measured.

### 2.9 Analysis of juxta-nuclear punctate structures

Cells were treated with siRNAs against SCFD1 for 48 h (see Section 2.3), followed by the SLTxB assay (Section 2.5). Cells were immunostained with antibodies against Rab6 (C-19, Santa Cruz Biotechnology, CA, United States). For this, cells were fixed with 3% PFA solution in PBS for 20 min at room temperature, followed by 5 min quenching with 30 mM glycine in PBS and three PBS washes. Cells were permeabilised for 5 min with 0.1% Triton X-100 (Sigma) in PBS, followed by three PBS washes. Then cells were incubated with primary antibody diluted in PBS for 1 h at room temperature, followed by three PBS washes and incubation with secondary antibody for 1 h at room temperature. After two PBS washes, nuclei were stained with 0.2 μg/ml Hoechst 33342 diluted in PBS for 5 min, followed by three further PBS washes. Coverslips were mounted with Mowiol on glass slides. Images were acquired on Olympus FluoView FV3000 microscope with a 60x PlanApo 1.4 NA oil immersion objective.

### 2.10 Statistical analysis

An ANOVA test with a Bonferroni post-hoc test was performed for the quantitative data from the Shiga-like toxin-1 B subunit functional assay. For the analysis of tubules in the live cell imaging data, either a paired two-sided student’s t-test or the Wilcoxon signed-rank test was performed. The Pearson correlation coefficient for the scatterplots was calculated with the corresponding function in Microsoft Excel.

## 3 Results

### 3.1 Systematic siRNA screen for molecular modulators of the Rab6-dependent pathway

**FIGURE 1:**
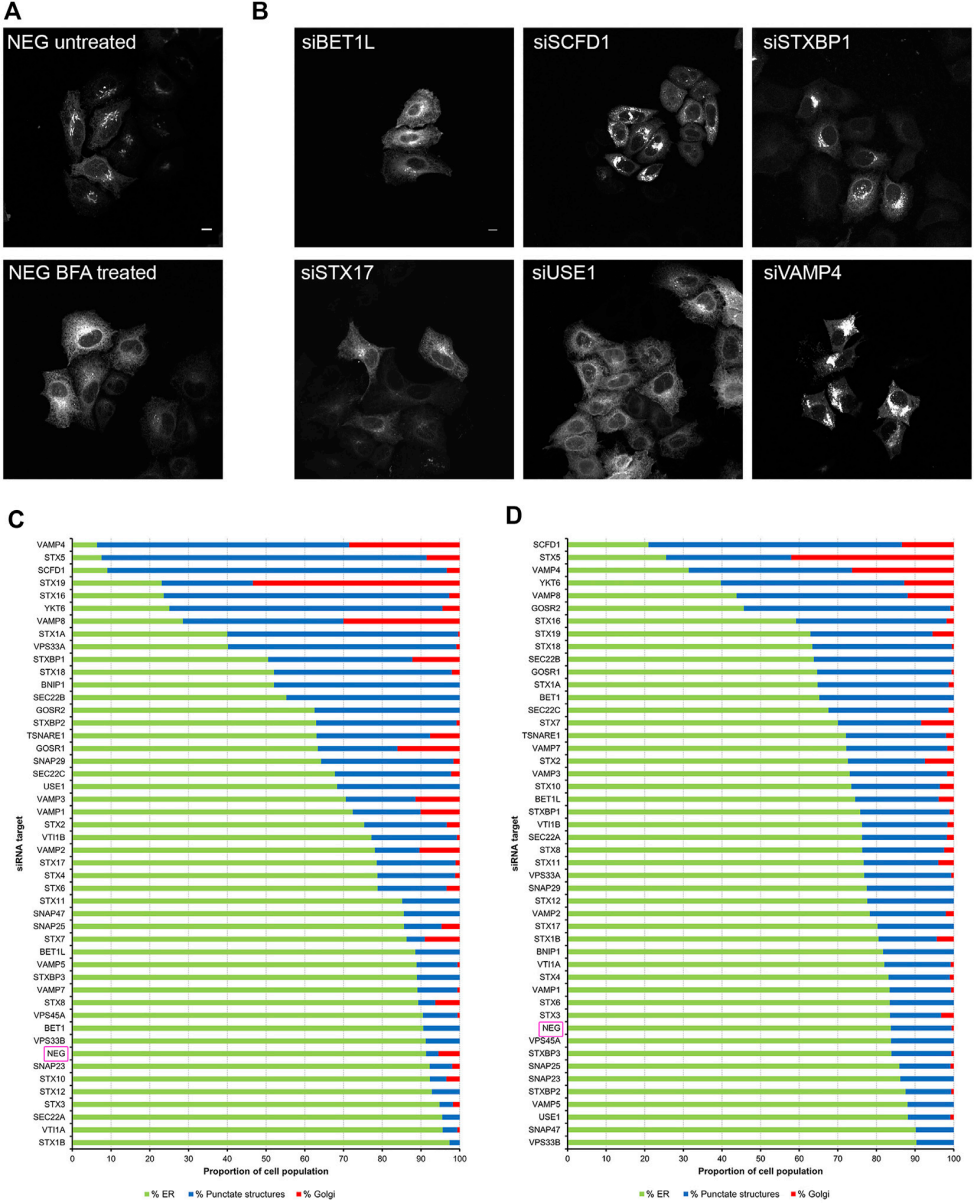
Golgi redistribution assay in EGFP-Rab6A transfected cells. Cells were transfected with siRNAs for 48 h, followed by transfection with either EGFP-Rab6A or EGFP-Rab6A′ constructs for 12 h. Cells were treated with 10 μg/ml BFA for 15 min at 37°C and then fixed and stained with 0.2 μg/ml Hoechst 33342. **(A)** Representative confocal images of siNEG-treated EGFP-Rab6A expressing HeLa Kyoto cells, either untreated or treated with BFA. Scale bar: 10 µm. **(B)** Representative confocal images of BFA treated EGFP-Rab6A expressing HeLa Kyoto cells incubated with various siRNAs against SNARE or SNARE-like proteins. Images represent different phenotypes observed. Scale bar: 10 µm. **(C)** Quantitative analysis of the EGFP-Rab6A redistribution assay. A minimum of 100 cells transfected with EGFP-Rab6A constructs were analysed per siRNA treatment (y-axis) and categorised into three phenotypic groups, namely ER (green bars)—cells displaying a high fluorescent signal in peripheral areas; punctate structures (blue bars)—cells with a distribution of fluorescent signals in juxta-nuclear punctate structures; and Golgi (red bars)—cells with an intense juxta-nuclear signal. The percentage of cells per treatment within each group is displayed on the x-axis. Data are represented as the mean of two independent experiments. **(D)** Quantitative analysis of the EGFP-Rab6A′ redistribution assay. A minimum of 100 cells transfected with EGFP-Rab6A′ constructs were analysed per siRNA treatment (y-axis) and categorised into three phenotypic groups, as for **(C)**. Data are represented as the mean of two independent experiments.

**FIGURE 2:**
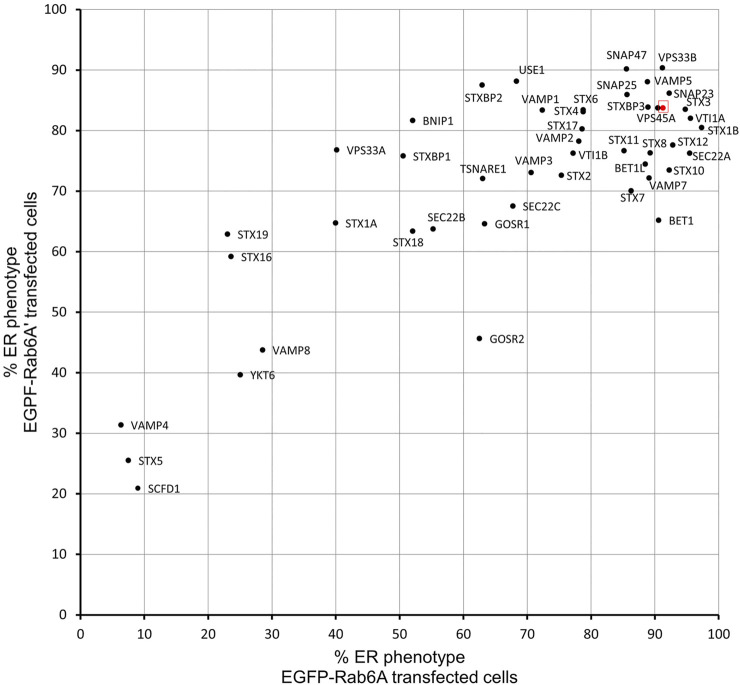
Comparison of EGFP-Rab6A and EGFP-Rab6A′ redistribution assays. Scatterplot showing the percentage of ER phenotypes in the population after each SNARE or SNARE-like protein depletion and BFA treatment in EGFP-Rab6A and EGFP-Rab6A′ expressing cells. The x-axis represents the percentage of ER phenotypes resulting from each depletion in EGFP-Rab6A transfected cells and the y-axis represents the percentage of ER phenotypes resulting from each depletion in EGFP-Rab6A′ transfected cells. The highlighted red dot represents the result from negative (NEG) control transfected cells. Pearson’s coefficient is 0.79.

We utilised a well-established assay, using the fungal metabolite brefeldin A (BFA) to induce the rapid relocation of Golgi markers to the endoplasmic reticulum (ER). In order to visualise this process, cells were initially transfected with DNA constructs encoding EGFP-Rab6A, thereby allowing us to monitor Rab6 itself directly. Under non-treated control conditions, or when cells were incubated with a non-targeting negative control small interfering RNA (siRNA) (NEG), Rab6A was seen in its characteristic subcellular pattern, mostly associated with juxta-nuclear Golgi membranes. Following 15 min of treatment with BFA, however, Rab6A was seen to have rapidly redistributed to the ER (Figure 1A). Cells were then treated with a library of siRNAs targeting SNARE and SNARE-related proteins. In each case, a pool of two independent siRNAs was used to achieve gene silencing (Supplementary Table S1). Following 48 h of down-regulation with the siRNAs, the cells were treated with BFA, as described above, revealing various phenotypes when the SNARE and SNARE-related proteins were depleted (Figure 1B). These phenotypes were visually examined, and the cell population was categorised into three groups: ER, punctate structures and Golgi (Figure 1C). As expected, more than 90% of NEG control cells exhibited complete redistribution of Rab6A to an ER pattern. However, 13 of the siRNA treatments showed an ER phenotype in less than 50% of the cell population, and of these, three siRNA treatments (VAMP4, STX5, and SCFD1) exhibited an ER redistribution phenotype in less than 10% of the cell population. Cells depleted of either VAMP4, STX5 or SCFD1 displayed a high proportion of the population containing punctate Rab6A structures. The depletion of a number of the genes resulted in little discernible change in the juxta-nuclear Rab6A signal in large populations of the cells; most notably, this was observed in cells treated with siRNAs targeting STX19. Rab6A exists in two splice variants, Rab6A and Rab6A′, so we repeated these BFA redistribution experiments examining the effects on the Rab6A′ variant. Overall, very similar results were obtained from cells transfected with EGFP-Rab6A′ expression constructs (Figure 1D). Comparison of the redistribution assays between the EGFP-Rab6A and EGFP-Rab6A′ transfected cells revealed a high correlation between the two isoforms, with a Pearson’s correlation coefficient of 0.79 (Figure 2). To confirm the efficiency of siRNA-mediated knockdown, a selected number of the experiments were subjected to qPCR analysis, using the same incubation period as for the BFA experiments. The knockdown efficiency of all siRNAs tested was found to be greater than 70% in all cases, with the exception of those targeting STX19 and VAMP4 (Supplementary Figure S1 and Supplementary Table S2).

### 3.2 Identification of differential modulators of the COPI-dependent and the Rab6-dependent retrograde pathways

**FIGURE 3:**
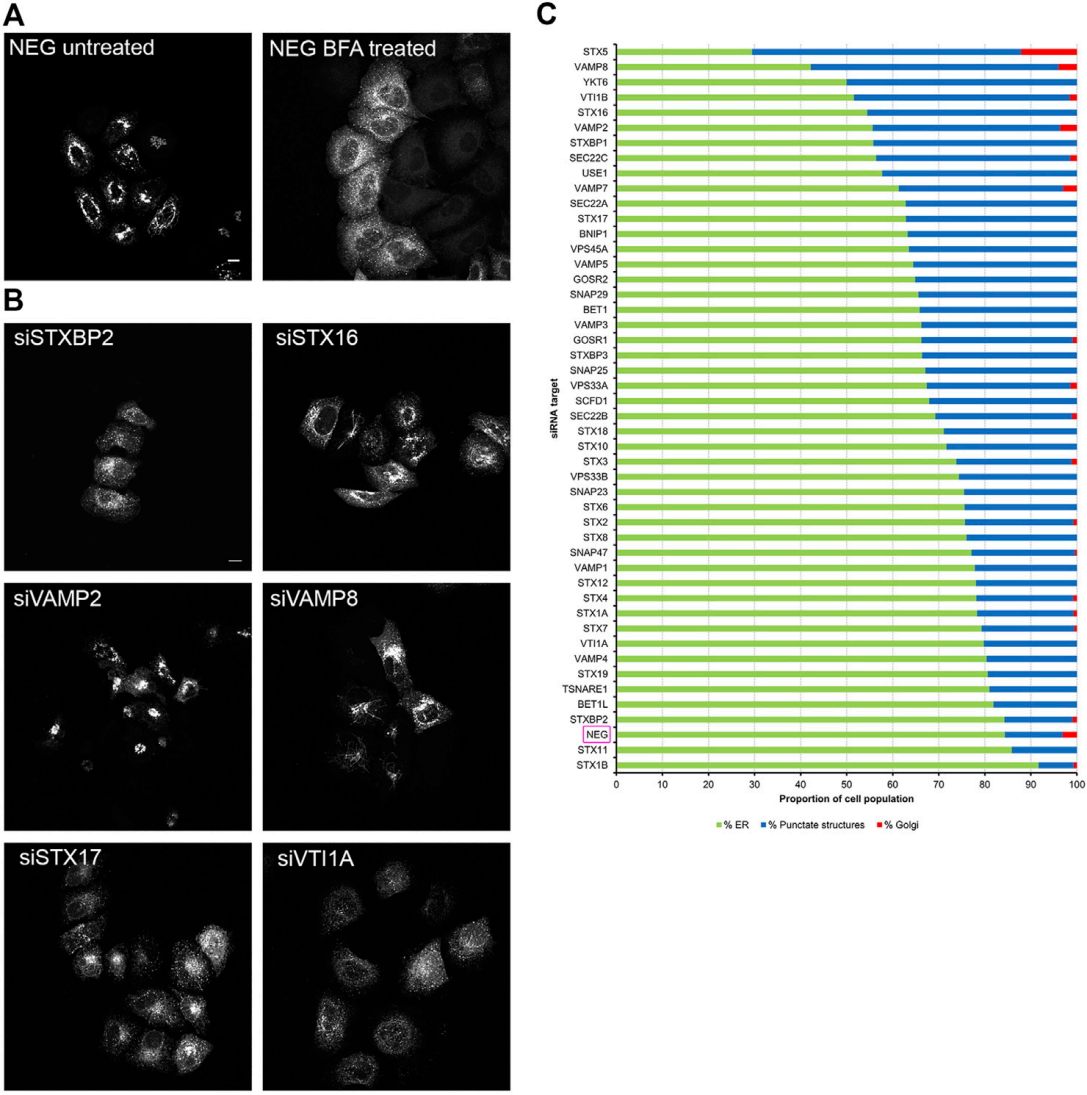
Golgi redistribution assay in EGFP-KDELR transfected cells. Cells were transfected with siRNAs for 48 h, followed by transfection with EGFP-KDELR constructs for 12 h. Cells were treated with 10 μg/ml BFA for 15 min at 37°C and then fixed and stained with 0.2 μg/ml Hoechst 33342. **(A)** Representative confocal images of siNEG-treated EGFP-KDELR expressing HeLa Kyoto cells, either untreated or treated with BFA. Scale bar: 10 µm. **(B)** Representative images of BFA treated EGFP-KDELR expressing HeLa Kyoto cells incubated with various siRNAs against SNARE or SNARE-like proteins. Images represent different phenotypes observed. Scale bar: 10 µm. **(C)** Quantitative analysis of the EGFP-KDELR redistribution assay. A minimum of 100 cells transfected with EGFP-KDELR constructs were analysed per siRNA treatment (y-axis) and categorised into three phenotypic groups, namely ER (green bars)—cells displaying a high fluorescent signal in peripheral areas; punctate structures (blue bars)—cells with a distribution of fluorescent signals in juxta-nuclear punctate structures; and Golgi (red bars)—cells with an intense juxta-nuclear signal. The percentage of cells per treatment within each group is displayed on the x-axis. Data are represented as the mean of two independent experiments.

**FIGURE 4:**
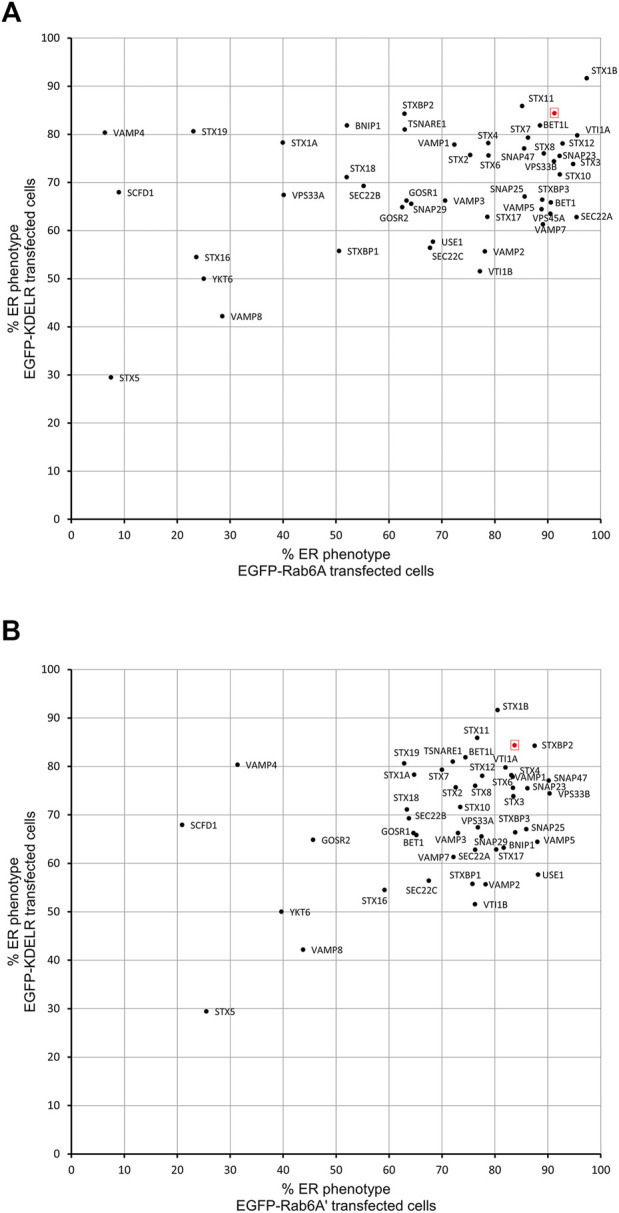
Comparison of Golgi redistribution assays. **(A)** Scatterplot showing the percentage of ER phenotypes in the population after depletion of each SNARE or SNARE-like protein and BFA treatment in EGFP-Rab6A and EGFP-KDELR expressing cells. The x-axis represents the percentage of ER phenotypes resulting from each depletion in EGFP-Rab6A transfected cells, and the y-axis represents the percentage of ER phenotypes resulting from each depletion in EGFP-KDELR transfected cells. The highlighted red dot represents the result from negative (NEG) control transfected cells. Pearson’s coefficient is 0.4. **(B)** Scatterplot showing the percentage of ER phenotypes in the population after depletion of each SNARE or SNARE-like protein and BFA treatment in EGFP-Rab6A′ and EGFP-KDELR expressing cells. The x-axis represents the percentage of ER phenotypes resulting from each depletion in EGFP-Rab6A′ transfected cells, and the y-axis represents the percentage of ER phenotypes resulting from each depletion in EGFP-KDELR transfected cells. The highlighted red dot represents the result from negative (NEG) control transfected cells. Pearson’s coefficient is 0.4.

In order to evaluate whether the various effects we had observed on Rab6A and Rab6A′ redistribution were more widespread with respect to other molecules moving between the Golgi and ER, we repeated the siRNA screen using a different class of protein. Specifically, we transfected cells with a construct encoding EGFP-tagged KDEL receptor (KDELR), a seven transmembrane domain protein, which plays a key role in cells retrieving ER residents that have been lost from that compartment (Figure 3). This approach would potentially allow us to identify Golgi-to-ER fusion machinery specific to carriers coated with Rab6A versus more general machinery. Similar to that seen in the Rab6A experiments, various phenotypes were visible in EGFP-KDELR transfected cells following siRNA incubation and BFA treatment. In NEG-treated cells, approximately 85% of the cell population was observed to have EGFP-KDELR in the ER following BFA treatment (Figure 3A). By contrast, the depletion of a number of the SNARE and SNARE-like proteins resulted in populations of cells with juxta-nuclear and punctate membrane structures remaining at the end of the experiment (Figure 3B). These were manually classified similar to that for Rab6A (Figure 3C). This revealed that the depletion of STX5 resulted in the strongest phenotype, with only 30% of the cell population showing an ER pattern of the EGFP-KDELR. Interestingly, EGFP-KDELR transfected cells depleted of either VAMP4 or SCFD1 showed noticeably milder redistribution phenotypes compared to those seen in EGFP-Rab6A/Rab6A′ transfected cells. Together, these results suggest that our approach was able to discriminate fusion machinery preferentially associated with the Rab6-dependent retrograde pathway. A comparison between the results from the various redistribution assays revealed a low Pearson’s correlation coefficient of 0.4 between the EGFP-Rab6A and EGFP-KDELR transfected cells (Figure 4A). A similar result was seen when comparing between the EGFP-Rab6A′ and EGFP-KDELR transfected cells (Figure 4B).

### 3.3 Analysis of Rab6-dependent retrograde cargo trafficking

**FIGURE 5:**
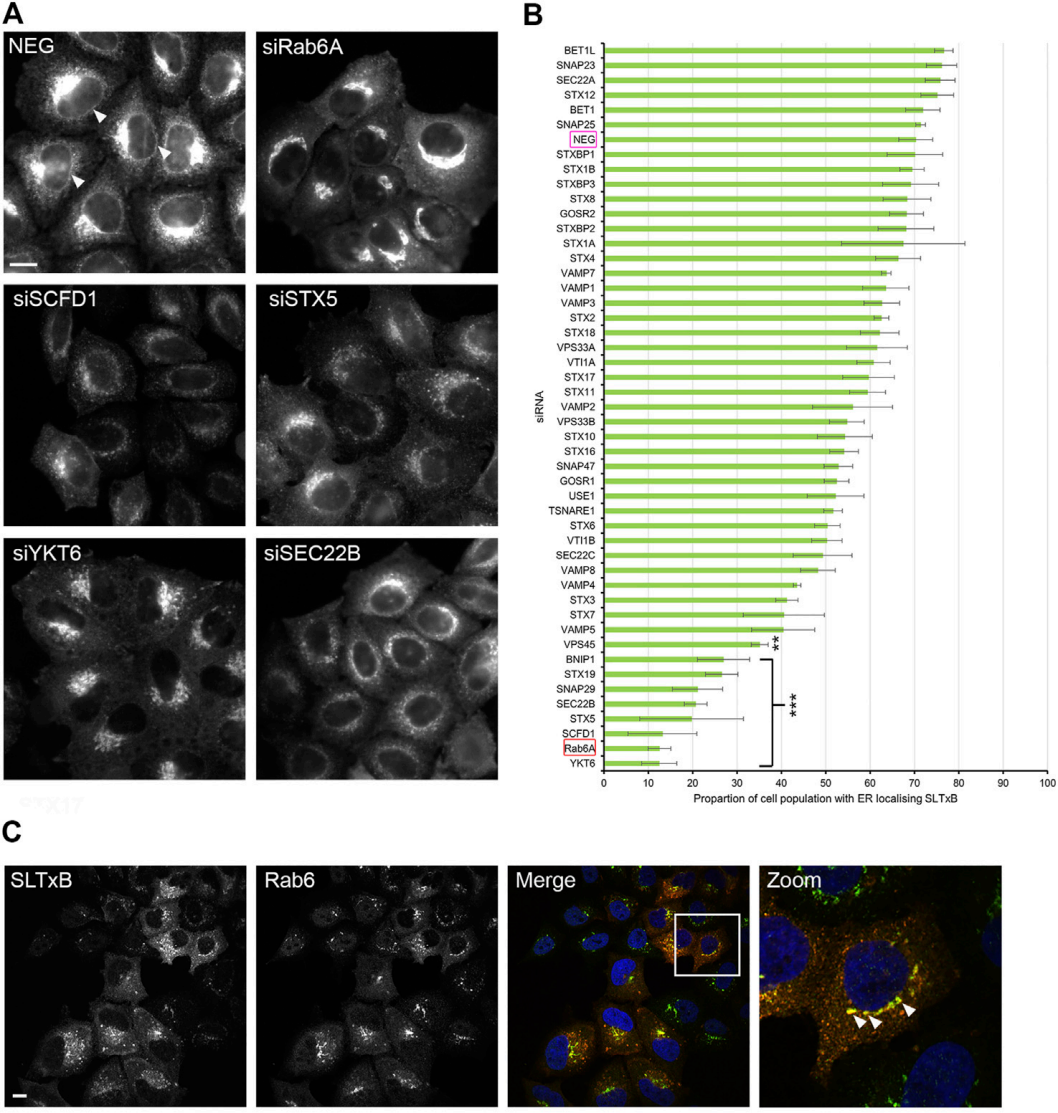
SLTxB trafficking assay. Cells were transfected with siRNAs for 48 h followed by incubation with SLTxB-Cy3 for 4 h at 37°C, and then fixed and stained with 0.2 μg/ml Hoechst 33342. **(A)** Representative images of negative control (NEG) and positive control (siRAB6A) cells as well as cells showing various SLTxB distributions that differed from those seen on control cells, following the siRNA treatments as indicated. Arrowheads highlight the nuclear envelope SLTxB signal, indicating toxin arrival at the ER. Scale bar: 10 µm. **(B)** Quantitative analysis of the SLTxB assay. The x-axis represents the percentage of the cell population with an ER localising SLTxB signal, and the y-axis represents each siRNA treatment. The magenta box highlights the negative control (NEG), and the red box highlights the positive control (Rab6A). Data are represented as the mean ± SEM of three independent experiments. An Anova test with a Bonferroni post-hoc test was performed against the NEG controls. *P* < 0.01 **, *p* < 0.001 ***. **(C)** Representative images of cells depleted for SCFD1, showing strong co-localisation between SLTxB (red) and Rab6 (green) Arrowheads highlight punctate structures positive for both SLTxB and Rab6.

**FIGURE 6:**
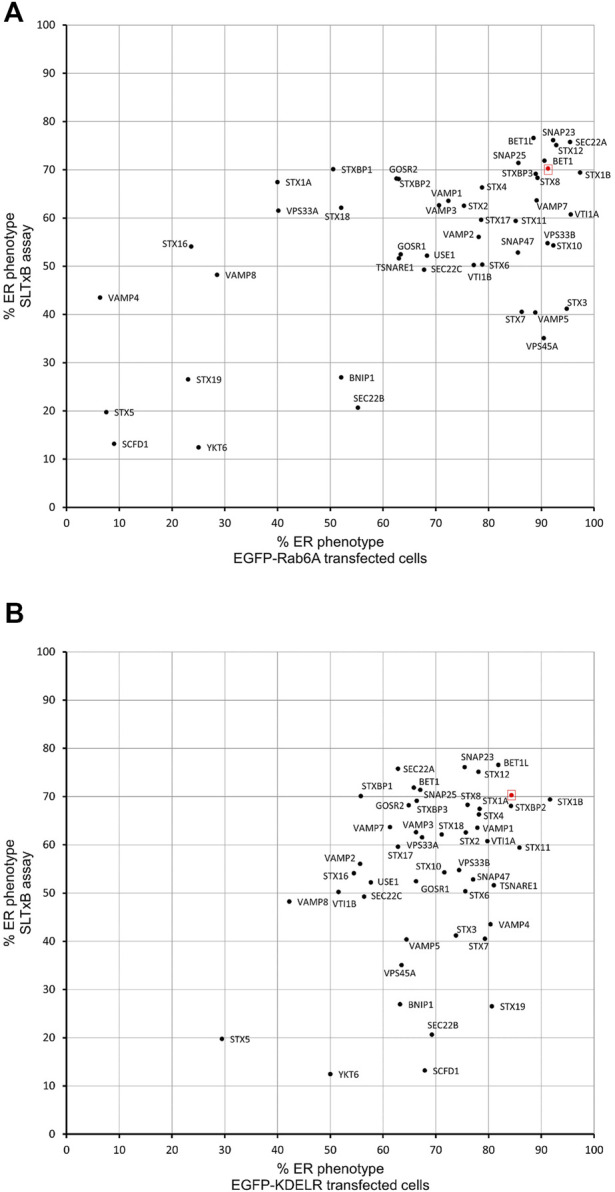
Comparison of SLTxB assay with Golgi redistribution assay. **(A)** Scatterplot showing the percentage of ER phenotypes in the population after depletion of each SNARE or SNARE-like protein in BFA treated EGFP-Rab6A expressing cells and each depletion in SLTxB treated cells. The x-axis represents the percentage of ER phenotypes resulting from each depletion in EGFP-Rab6A transfected cells, and the y-axis represents the percentage of ER phenotypes resulting from each depletion in SLTxB treated cells. The highlighted red dot represents the result from negative (NEG) control transfected cells. Pearson’s coefficient is 0.46. **(B)** Scatterplot showing the percentage of ER phenotypes in the population after depletion of each SNARE or SNARE-like protein in BFA treated EGFP-KDELR expressing cells and each depletion in SLTxB treated cells. The x-axis represents the percentage of ER phenotypes resulting from each depletion in EGFP-KDELR transfected cells, and the y-axis represents the percentage of ER phenotypes resulting from each depletion in SLTxB treated cells. The highlighted red dot represents the result from the negative (NEG) control transfected cells. Pearson’s coefficient is 0.21.

The experiments described above suggested that siRNA screening was able to identify membrane fusion machinery that was preferentially associated with Rab6A carriers. To further validate our approach, we decided to assess a retrograde cargo, the Shiga-like toxin B-chain (SLTxB), which has been shown previously to be exclusively carried by Rab6 as it traffics to the ER. Cells were treated with siRNAs against SNARE and SNARE-related proteins for 48 h and then incubated with Cy3-labelled SLTxB for 4 h. Under control (NEG) conditions, SLTxB-Cy3 transits the retrograde pathway, eventually arriving in the ER, where it can be clearly distinguished by its visualisation in the nuclear envelope (Figure 5A, arrowheads). The siRNA-mediated depletion of a number of membrane fusion machinery molecules resulted in SLTxB failing to reach the ER. The strongest effects were seen in cells depleted for either STX5, SCFD1 or YKT6 (Figure 5A). Quantification of the cell population in these three experimental conditions revealed that, on average, only between 12% and 20% of the cells showed an ER localising SLTxB signal. This figure was similar to that seen for the depletion of Rab6A itself, the positive control used in this experiment (Figure 5B). Furthermore, immunostaining revealed that a large proportion of the remaining SLTxB-positive punctate structures in siSCFD1-treated cells were coated with Rab6 (Figure 5C). Correlation analysis between the ER redistribution of EGFP-Rab6A and ER delivery of SLTxB produced a relatively high Pearson coefficient of 0.46 (Figure 6A). Of particular note was the observation that depletion of SCFD1 produced the strongest phenotype in both of these assays. By contrast, the Pearson coefficient between the redistribution assay in EGFP-KDELR-transfected cells and the SLTxB assay was only 0.21 (Figure 6B). This supports the notion that the assays, using a variety of different molecule types, are able to discriminate machinery that is preferentially associated with the Rab6 retrograde pathway.

Rab6A exists in two different splice variants, Rab6A and Rab6A′, differing by three amino acids. Previous work has suggested that there are functional differences between Rab6A and Rab6A′, with Rab6A′ implicated in retrograde transport from recycling endosomes to the Golgi (Mallard et al., 2002; Del Nery et al., 2006). We therefore co-transfected cells with GFP-Rab6A and mCh-Rab6A′ constructs and treated the cells with BFA for 15 min, similar to the method used in our screen. We then measured the co-occurence of GFP-Rab6A and mCh-Rab6A′ on membrane tubules. There was a high degree of co-occurrence (>90%) of GFP-Rab6A and mCh-Rab6A′ on tubules, indicating that our transport assay was not discriminating between Rab6A and Rab6A′ carriers (Supplementary Figures S2). This finding was further supported when we examined the correlation of redistribution of EGFP-Rab6A′ and ER delivery of SLTxB (Supplementary Figures S3). The strongest candidates observed were the same as those seen in the EGFP-Rab6A studies, and the Pearson coefficient between the redistribution of EGFP-Rab6A’ and ER delivery of SLTxB was found to be 0.36.

### 3.4 A role for SCFD1 in the Rab6-dependent retrograde pathway

**FIGURE 7:**
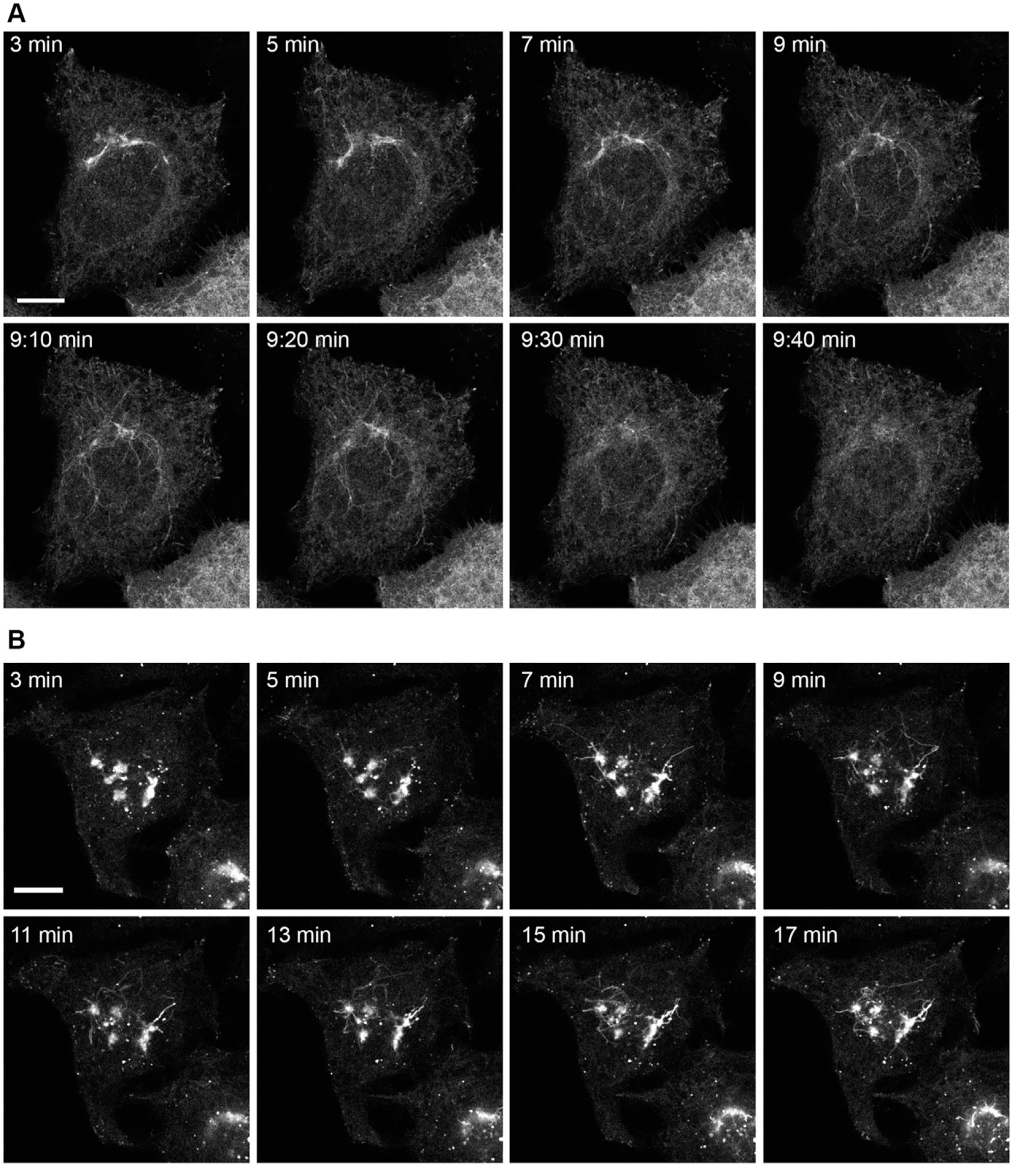
Time-lapse imaging of EGFP-Rab6A redistribution after BFA treatment in siSCFD1 treated cells. HeLa Kyoto cells were transfected with siRNAs for 48 h and EGFP-Rab6A constructs for 12 h and treated with 10 μg/ml BFA. Confocal time-lapse images were taken every 10 s. **(A)** Example frames from a time-lapse movie in control cells (NEG). The time points refer to the time after BFA addition. **(B)** Example frames from a time-lapse movie in cells treated with siRNAs against SCFD1. The time points refer to the time after BFA addition. Scale bars: 10 µm.

Given our findings that the depletion of SCFD1 consistently resulted in the failure of cells to redistribute EGFP-Rab6A/Rab6A’ under BFA conditions and that SLTxB was unable to reach the ER, whereas KDELR was comparatively less affected, we decided to focus on this particular fusion machinery molecule. In order to better understand the potential role of SCFD1 in the Rab6-dependent pathway, a series of live-cell experiments were performed on individual cells. Specifically, we were interested in deepening our understanding of the nature of the Rab6A retrograde carriers and their properties in the presence and absence of SCFD1 (Figure 7). Cells over-expressing EGFP-Rab6A were treated with BFA and subjected to time-lapse imaging. In control (NEG) cells, membrane tubules emanating from the Golgi apparatus were clearly visible within 3 min of BFA treatment (Figure 7A). These tubules were highly dynamic in nature, and by less than 10 min after BFA addition, they were no longer visible, and the ER could be clearly seen (Figure 7A). By contrast, in SCFD1-depleted cells, the initial membrane tubules that formed following BFA treatment were shorter than those seen in control cells. However, these tubules persisted in cells for an extensive period of time, and during the course of the experiment, the juxta-nuclear Rab6A signal was never completely redistributed (Figure 7B). These observations were consistent with those seen in the siRNA screen (Figure 1B). These time-lapse experiments were then repeated in cells expressing EGFP-KDELR. Following BFA treatment, long membrane tubules were first seen in control (NEG) cells within approximately 3 min of BFA addition, and the Golgi was seen to redistribute to the ER and small punctate structures within a few minutes (Supplementary Figures S4A). In cells depleted for SCFD1, the tubules emerged at a similar time to that seen in control cells, however, the redistribution of the Golgi seemed slower (Supplementary Figures S4B).

**FIGURE 8:**
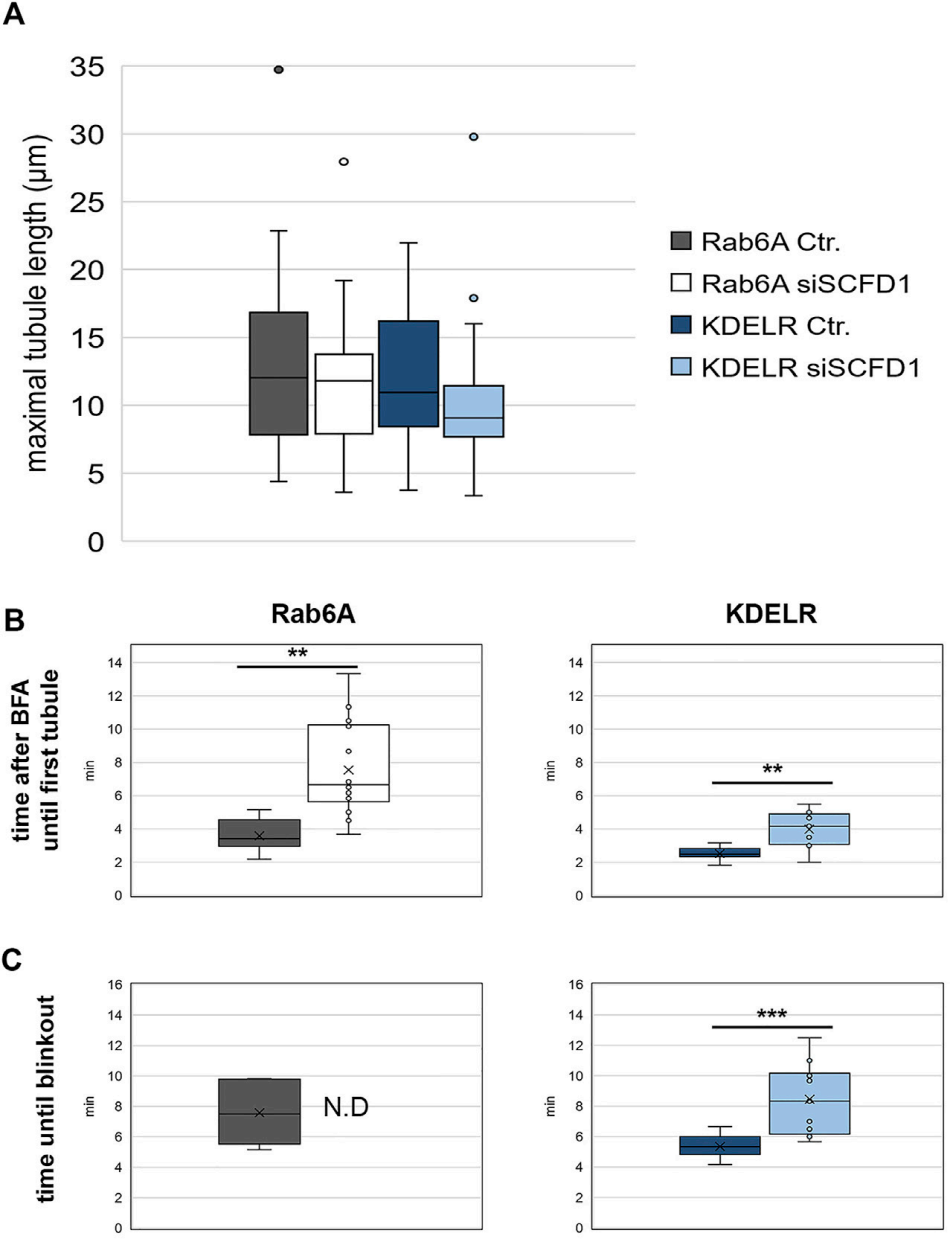
Quantitative analysis of EGFP-Rab6A and EGFP-KDELR tubules after BFA treatment. HeLa Kyoto cells were transfected with siRNAs for 48 h and then transfected with constructs encoding either EGFP-Rab6A or EGFP-KDELR for 12 h and treated with 10 μg/ml BFA. Confocal time-lapse images were taken every 10 s for a total of 15 min or until the redistribution of the Golgi marker to the ER was complete within this time. **(A)** Maximal tubule length for negative control (NEG) and siSCFD1 treated EGFP-Rab6A or EGFP-KFDELR expressing cells. The y-axis represents the maximal tubule length (µm). **(B)** Quantitative analysis of the time after BFA treatment until the first tubule was visible in negative control (NEG) and siSCFD1 treated EGFP-Rab6A and EGFP-KDELR expressing cells. The y-axis represents the time after BFA treatment until the first tubule was observed (min). **(C)** Quantitative analysis of the time after BFA treatment until full ER redistribution (blinkout) of the Golgi marker occurred in negative control (NEG) and siSCFD1 treated EGFP-Rab6A and EGFP-KDELR expressing cells. The y-axis represents the time after BFA treatment until blinkout (min). A paired two-sided student’s t-test was performed. *P* < 0.01 **, *p* < 0.01 ***.

In order to characterise the phenotypes seen in the cells, we repeated these time-lapse experiments and quantified the properties of the membrane tubules. We initially measured the mean length of both the EGFP-Rab6A and EGFP-KDELR tubules, under control (NEG) and SCFD1-depleted conditions. This revealed that their lengths were on average between 9 µm and 12 μm, but that there was no significant difference between the lengths of the Rab6A and KDELR tubules, or between the tubules generated in control versus siRNA-treated cells (Figure 8A). We next examined the kinetics of tubule biogenesis. Analysis of the time for the emergence of the first tubule after BFA treatment revealed that for both Rab6A and KDELR tubules that this occurred within 2–4 min of BFA addition. In both cases, however, the SCFD1 depletion resulted in a delay in tubule emergence. This was more profound in the case of the EGFP-Rab6A tubules (Figure 8B). Finally, we examined the ability of the tubules to redistribute either the Rab6A or the KDELR to the ER. This phenomenon is known as Golgi blinkout. For Rab6A, under control (NEG) conditions, the mean blinkout time was determined at 10 min after BFA addition. Strikingly, however, in the cells depleted for SCFD1, blinkout was never achieved up to the maximum imaging period of the experiment. The depletion of SCFD1 resulted in a delayed blinkout of the KDELR, but this delay was less severe than that seen for Rab6A (Figure 8C).

## 4 Discussion

Although the Rab6-dependent retrograde pathway, operating between the Golgi and ER, was discovered more than 20 years ago (Girod et al., 1999; White et al., 1999), many questions relating to its molecular regulation remain unanswered (Heffernan and Simpson, 2014). In this study, we utilised a systematic siRNA screening approach to examine the role of SNARE and SNARE-related proteins in the Rab6-dependent retrograde pathway, in an attempt to identify membrane fusion machinery associated with this pathway (Wang et al., 2017).

Rab6A exists in two variants, Rab6A and Rab6A′, which differ by just three amino acids. Using a well-established method for rapidly invoking the redistribution of Golgi membranes to the ER, we screened for factors that might inhibit this process. Although others have shown functional differences between the two variants (Mallard et al., 2002; Del Nery et al., 2006), in our screens we obtained similar redistribution results, also consistent with our observations of high levels of co-occurrence of Rab6A and Rab6A′ on the same tubular intermediates. The depletion of three candidates, SCFD1, STX5, and VAMP4, consistently resulted in the failure of membranes coated with both Rab6A isoforms to redistribute to the ER. Of these three, SCFD1, also known as SLY1, a Sec1/Munc18 (SM) protein, produced the strongest phenotype. Depletion of SCFD1 not only produced a strong trafficking defect in the Rab6A/Rab6A′ redistribution assays but also in an independent assay measuring the delivery of a Rab6A cargo molecule, SLTxB, to the ER. However, depletion of SCFD1 had only a mild effect on the redistribution of the KDELR, a known cargo of the COPI coat machinery, to the ER under BFA conditions. SCFD1 was first identified in yeast as an interacting partner of Ypt1 (Rab1 in mammalian cells) (Dascher et al., 1991). SCFD1 exists in both a cytosolic and Golgi pool and has also been shown to be present at ER exit sites (ERES) (Nogueira et al., 2014). SCFD1 interacts with several SNARE proteins, including STX5 (syntaxin-5), another molecule that produced a strong phenotype on Rab6A/Rab6A′ redistribution in our screen. The interaction of SCFD1 with STX5 has been proposed to have many regulatory roles, including a role in anterograde trafficking from the ER to the Golgi (Dascher and Balch, 1996; Williams et al., 2004), *via* a high affinity interaction at the N-terminus of STX5 (Grabowski and Gallwitz, 1997; Bracher and Weissenhorn, 2002; Yamaguchi et al., 2002). This interaction causes a conformational change in the C-terminal ß-strand of SCFD1 (Araç et al., 2005). SCFD1 can also bind the closed confirmation of STX5, thus causing a rearrangement of STX5 into an open conformation. This, in turn, stimulates the assembly of an ER-Golgi SNARE complex consisting of Sed5-Bos1-BET1-Sec22 (Demircioglu et al., 2014). Our screen found that depletion of STX5 not only inhibited the trafficking of Rab6A/Rab6A’ and its cargo SLTxB to the ER, but also the COPI cargo KDELR. This implicates a role for STX5 in both Golgi-to-ER retrograde pathways.

Indeed a role for STX5 in Golgi-to-ER retrograde trafficking is further supported by work from Smith and colleagues, who showed that STX5 is important for the retrograde transport of the subtilase cytotoxin in a COPI- and Rab6-dependent manner (Smith et al., 2009). Further links between Rab6 and STX5 are mediated through interaction with the COG complex (Shestakova et al., 2007; Fukuda et al., 2008; Kudlyk et al., 2013), a complex well-known to function in retrograde trafficking (Zolov and Lupashin, 2005). Other studies have previously linked STX5 function to Shiga toxin trafficking, although in the context of the plasma membrane to TGN transport step (Amessou et al., 2007).

Our results, however, also suggest a more specific role for SCFD1 in the Rab6-dependent pathway. Previous work in yeast has suggested that SCFD1 may function in the retrograde Golgi-to-ER pathway. The expression of a mutated Sly1p protein resulted in mis-sorting of the KDEL-motif-containing chaperone protein Kar2p (BiP in mammalian cells), as well as failure to relocate Sed5p (STX5) and Rer1p (Li et al., 2005). However, Nogueira and colleagues more recently showed that siRNA-mediated depletion of SCFD1/SLY1 had no effect on the retrograde trafficking of BiP (Nogueira et al., 2014), aligning with our own findings on KDELR redistribution, and further suggesting its major role is in the Rab6-dependent pathway.

SCFD1/SLY1 is a Sec1/Munc18 (SM) protein, which regulates the fusion activity of SNARE proteins. As such, it must interact with specific SNAREs, including any associated with Rab6-dependent Golgi-to-ER transport. Examination of the data from our screen assessing delivery of the Rab6 cargo, SLTxB, to the ER revealed that the strongest phenotype was seen on depletion of the v-SNARE YKT6. YKT6 has previously been identified to cooperate with STX5 in two distinct SNARE complexes, namely STX5-YKT6-GOSR1-BET1 (Zhang and Hong, 2001) and STX5-BET1L-GOSR1-YKT6 (Tai et al., 2004). Furthermore, mutational analysis of a yeast Sly1p protein that is deficient in binding Sed5p showed that Sly1p could bind to YKT6 (Peng and Gallwitz, 2004). Additionally, YKT6 and STX5 localise to the Golgi (Dascher et al., 1994; Volchuk et al., 2004), making YKT6 a candidate for loading on to Rab6 carriers destined for the ER. Moreover, Klausner and colleagues have previously shown that YKT6 itself redistributes to the ER when cells are treated with BFA (Klausner et al., 1992). Given that our data revealed that SCFD1/SLY1 depletion had little effect on the production or length of BFA-induced Rab6A tubules but rather on their fusion, this strongly suggests that the fusion event at the ER is regulated by SCFD1 and YKT6.

Taken together, our results suggest that SCFD1 plays a critical role in the Rab6-dependent retrograde pathway. Additional likely components of the fusion machinery of Rab6 carriers are STX5 and YKT6, as both showed retrograde trafficking defects when depleted in cells, and both proteins have been shown by others to interact with SCFD1 (Grabowski and Gallwitz, 1997; Bracher and Weissenhorn, 2002; Yamaguchi et al., 2002; Peng and Gallwitz, 2004; Araç et al., 2005; Demircioglu et al., 2014). Our systematic siRNA screening approach has, for the first time, shed new light on the fusion machinery used in the Rab6-dependent pathway, but further studies will now be needed to identify the full range of interactors used on this pathway.

## Data Availability

The original contributions presented in the study are included in the article/Supplementary Material, further inquiries can be directed to the corresponding author.
