# Supplementary material for: An imaging-based RNA interference screen for modulators of the Rab6-mediated Golgi-to-ER pathway in mammalian cells
Source: Front Cell Dev Biol. 2022 Nov 29;10:1050190. doi: 10.3389/fcell.2022.1050190 (PMC9745180; doi:10.3389/fcell.2022.1050190)
Supplement: Supplementary file 1 [file DataSheet1.docx]

***Supplementary Material***

# Supplementary Figures and Tables

## Supplementary Figures


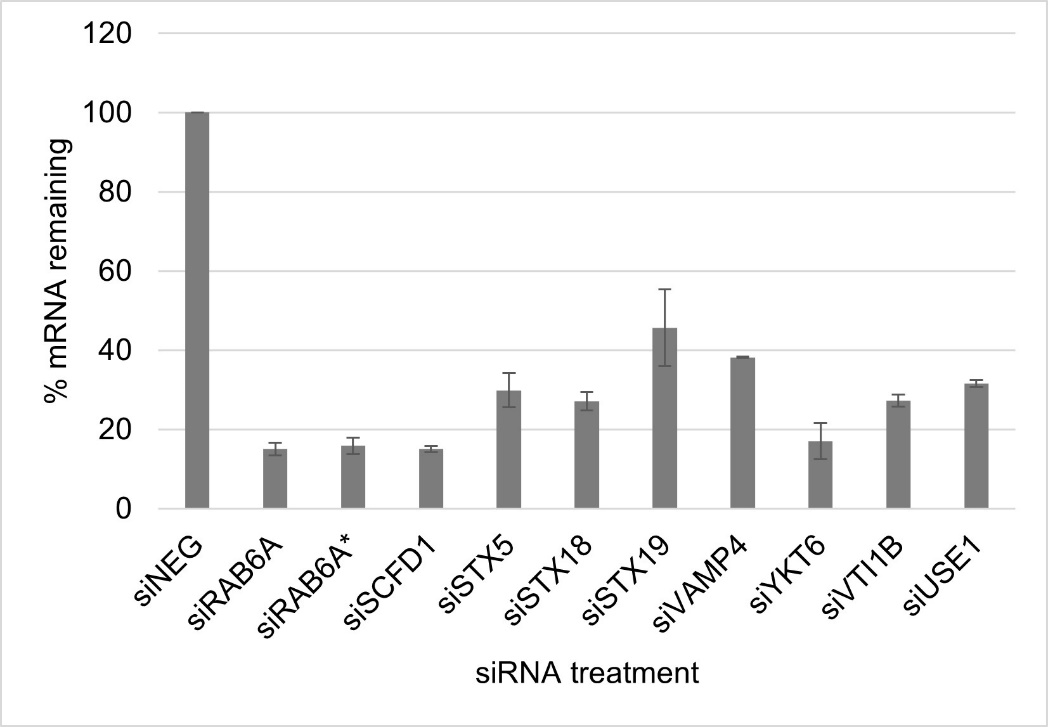


**Supplementary Figure S1: siRNA knockdown efficiency of selected targets.** Cells were treated for 48 h with siRNAs. Total RNA was extracted, and cDNA was synthesised, followed by qPCR analysis. In the sample labelled Rab6A*, qPCR primers detecting the Rab6A’ variant were used. Data are represented as the mean ± SEM of three independent experiments.


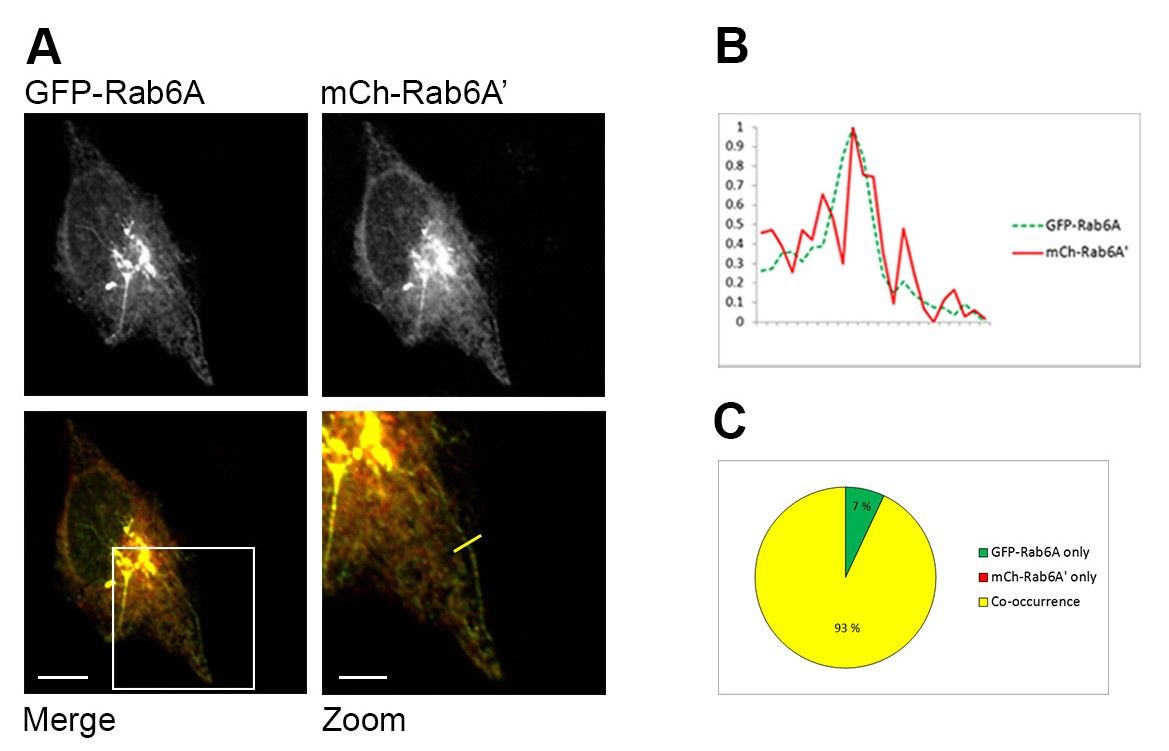


**Supplementary Figure S2: Co-occurrence of Rab6A and Rab6A’ on BFA-induced retrograde tubules.** (A) HeLa Kyoto cells co-expressing GFP-Rab6A (green) and mCh-Rab6A’ (red) following a 4 min BFA treatment. Scale bar: 10 μm. Zoom box, 30 μm x 30 μm. Zoomed section of the merge image with a 1.5 μm analysis line. Scale bar: 5 μm. (B) Intensity profiles plotted of both channels along the analysis line. Only intensities in both channels of 70% or higher with the same intensity peak position were defined as representative tubular structures containing co-occurring signals. (C) The percentage abundance of GFP-Rab6A/mCh-Rab6A’ positive tubules. A minimum of 90 tubules from typically 15 cells were analysed.


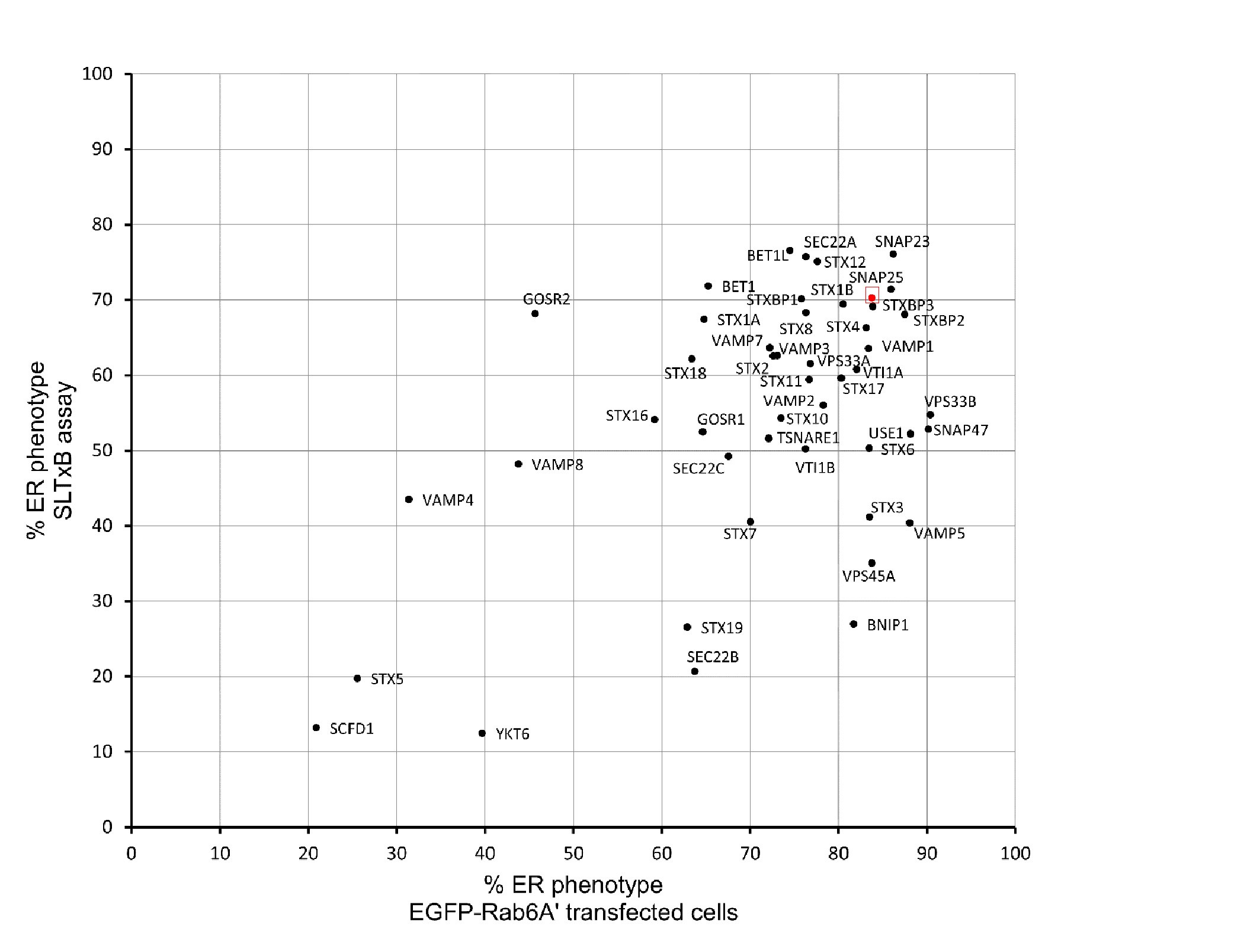


**Supplementary Figure S3: Comparison of SLTxB assay with Golgi redistribution assay in EGFP-Rab6A’ transfected cells.** Scatterplot showing the percentage of ER phenotypes in the population after depletion of each SNARE or SNARE-like protein in BFA treated EGFP-Rab6A’ expressing cells and each depletion in SLTxB treated cells. The x-axis represents the percentage of ER phenotypes resulting from each depletion in EGFP-Rab6A’ transfected cells, and the y-axis represents the percentage of ER phenotypes resulting from each depletion in SLTxB treated cells. The highlighted red dot represents the result from negative (NEG) control transfected cells. Pearson's coefficient is 0.36.


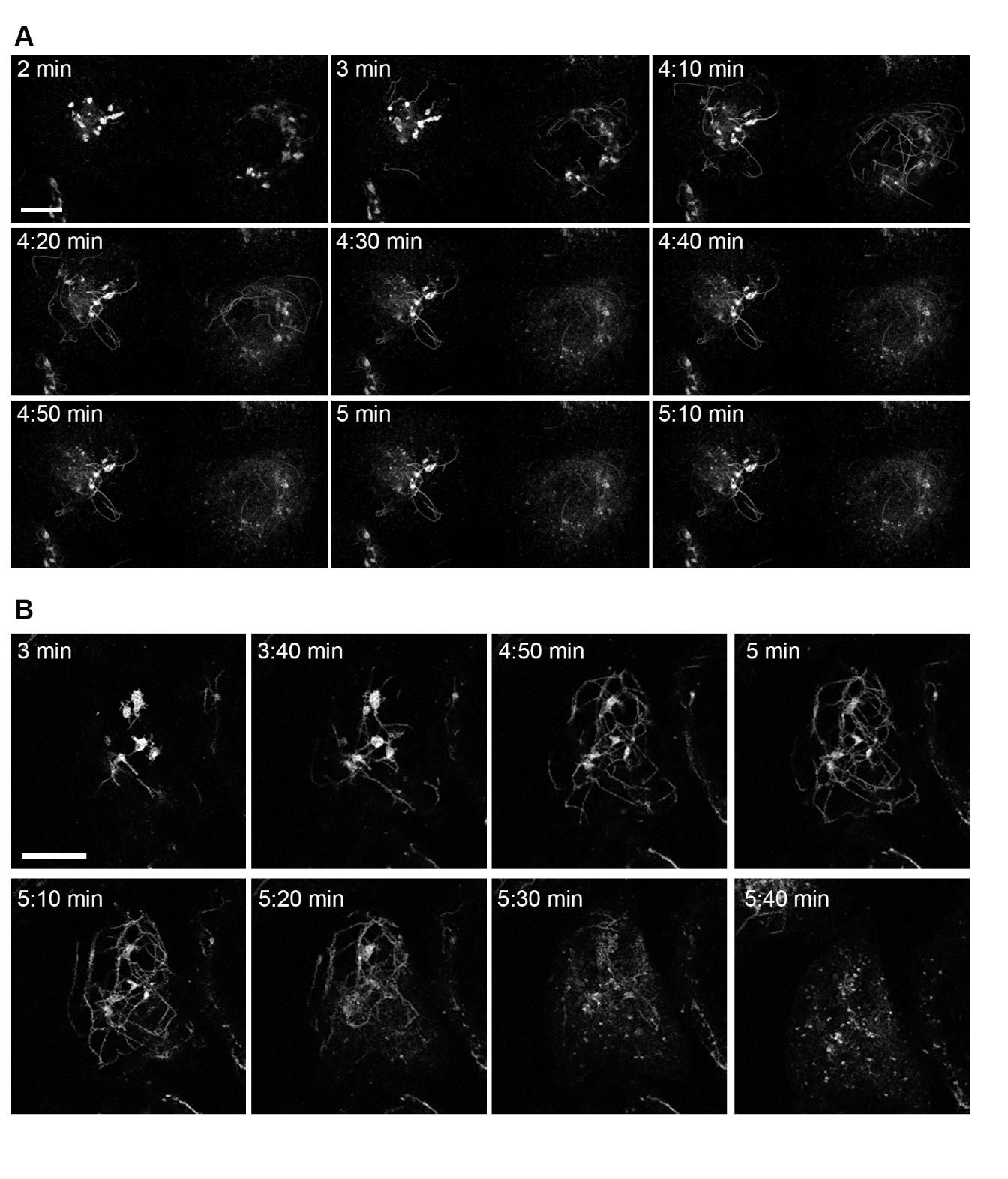


**Supplementary Figure S4: Time-lapse imaging of EGFP-KDELR redistribution after BFA treatment in siSCFD1 treated cells.** HeLa Kyoto cells were transfected with siRNAs for 48 h followed by constructs encoding EGFP-KDELR for 12h, and then treated with 10 µg/mL BFA. Confocal time-lapse images were taken every 10 sec. (A) Example frames from a time-lapse movie in control cells (NEG). The time points refer to the time after BFA addition. (B) Example frames from a time-lapse movie in cells treated with siRNAs against SCFD1. The time points refer to the time after BFA addition. Scale bars: 10 µm.

## Supplementary Tables

**Supplementary Table S1: List of siRNAs used.**

| **Gene** | **Name** | **Gene ID** | **siRNA ID 1** | **siRNA ID 2** |
| --- | --- | --- | --- | --- |
| STX1A | syntaxin 1A | 6804 | s13589 | s13590 |
| STX1B | syntaxin 1B | 112755 | s223220 | s41367 |
| STX2 | syntaxin 2 | 2054 | s4755 | s4756 |
| STX3 | syntaxin 3 | 6809 | s13592 | s13593 |
| STX4 | syntaxin 4 | 6810 | s13595 | s13596 |
| STX5 | syntaxin 5 | 6811 | s13598 | s13599 |
| STX6 | syntaxin 6 | 10228 | s19958 | s19959 |
| STX7 | syntaxin 7 | 8417 | s15978 | s15979 |
| STX8 | syntaxin 8 | 9482 | s18182 | s18183 |
| STX10 | syntaxin 10 | 8677 | s16534 | s16535 |
| STX11 | syntaxin 11 | 8676 | s16531 | s16532 |
| STX12 | syntaxin 12 | 23673 | s24307 | s24308 |
| STX16 | syntaxin 16 | 8675 | s16528 | s16529 |
| STX17 | syntaxin 17 | 55014 | s29992 | s29993 |
| STX18 | syntaxin 18 | 53407 | s28733 | s28734 |
| STX19 | syntaxin 19 | 415117 | s53949 | s53950 |
| TSNARE1 | t-SNARE domain containing 1 | 203062 | s47463 | s47464 |
| SNAP23 | synaptosomal-associated protein, 23kDa | 8773 | s16708 | s16709 |
| SNAP25 | synaptosomal-associated protein, 25kDa | 6616 | s13188 | s13189 |
| SNAP29 | synaptosomal-associated protein, 29kDa | 9342 | s17859 | s17860 |
| SNAP47 | synaptosomal-associated protein, 47kDa | 116841 | s225514 | s42059 |
| VAMP1 | vesicle-associated membrane protein 1 (synaptobrevin 1) | 6843 | s13664 | s13665 |
| VAMP2 | vesicle-associated membrane protein 2 (synaptobrevin 2) | 6844 | s13667 | s13668 |
| VAMP3 | vesicle-associated membrane protein 3 (cellubrevin) | 9341 | s17856 | s17857 |
| VAMP4 | vesicle-associated membrane protein 4 | 8674 | s16525 | s16526 |
| VAMP5 | vesicle-associated membrane protein 5 (myobrevin) | 10791 | s21198 | s21199 |
| VAMP7 | vesicle-associated membrane protein 7 | 6845 | s13670 | s13671 |
| VAMP8 | vesicle-associated membrane protein 8 (endobrevin) | 8673 | s16522 | s16523 |
| YKT6 | YKT6 v-SNARE homolog (S. cerevisiae) | 10652 | s20936 | s20937 |
| SEC22A | SEC22 vesicle trafficking protein homolog A (S. cerevisiae) | 26984 | s25658 | s25659 |
| SEC22B | SEC22 vesicle trafficking protein homolog B (S. cerevisiae) (gene/pseudogene) | 9554 | s18347 | s18348 |
| SEC22C | SEC22 vesicle trafficking protein homolog C (S. cerevisiae) | 9117 | s17398 | s17399 |
| BET1 | blocked early in transport 1 homolog (S. cerevisiae) | 10282 | s20092 | s20093 |
| BET1L | blocked early in transport 1 homolog (S. cerevisiae)-like | 51272 | s226724 | s226725 |
| GOSR2 | golgi SNAP receptor complex member 2 | 9570 | s18383 | s18384 |
| GOSR1 | golgi SNAP receptor complex member 1 | 9527 | s18278 | s18279 |
| VTI1A | vesicle transport through interaction with t-SNAREs homolog 1A (yeast) | 143187 | s225671 | s44510 |
| VTI1B | vesicle transport through interaction with t-SNAREs homolog 1B (yeast) | 10490 | s20556 | s20557 |
| USE1 | unconventional SNARE in the ER 1 homolog (S. cerevisiae) | 55850 | s226873 | s226874 |
| BNIP1 | BCL2/adenovirus E1B 19kDa interacting protein 1 | 662 | s2053 | s2054 |
| RAB6A | RAB6A, member RAS oncogene family | 5870 | s11684 | s11685 |
| STXBP1 | syntaxin binding protein 1 | 6812 | s13601 | s13602 |
| STXBP2 | syntaxin binding protein 2 | 6813 | s13604 | s13605 |
| STXBP3 | syntaxin binding protein 3 | 6814 | s13607 | s13608 |
| VPS33A | vacuolar protein sorting 33 homolog A (S. cerevisiae) | 65082 | s35195 | s35196 |
| VPS33B | vacuolar protein sorting 33 homolog B (yeast) | 26276 | s25352 | s25353 |
| VPS45 | vacuolar protein sorting 45 homolog (S. cerevisiae) | 11311 | s22295 | s22296 |
| SCFD1 | sec1 family domain containing 1 | 23256 | s23410 | s23411 |

**Supplementary Table S2: Details of qPCR primers used to assess knock-down efficiency.**

| **Target** | **Left primer sequence (5’-3’)** | **Right primer sequence (5’-3’)** |
| --- | --- | --- |
| RAB6A | ATGTACTTGGAGGATCGAACAGT | CAAGCTCCTGAACCGCTCT |
| RAB6A | GGATACTGCGGGTCAGGAG | TCCACTTTGTAGTTTGCTGGAA |
| SCFD1 | CTCGTGGGAGCCAACATG | CATACGCTTCAAAGCCACTGT |
| STX5 | GTCTTTGGTCGGGTTTCG | AGACGCATAACCTCGGACTC |
| STX18 | TTCTTCTGGAACACAGGAAAGAT | TCTGGGCATCCTGGTCTATC |
| STX19 | AACAATTTGGCAGATAATGTTCAA | TCATTGAAGCCACCAGACTTT |
| USE1 | TCCCTGATCAATTTCTTCTTTTCT | TGTCATCAAGAAGGACAACCA |
| VAMP4 | AAGTGAAAGGAGAAATCTTTTGGA | GGTCCAGATGGTCCCCTTAG |
| VTI1B | GGTCCAGATGGTCCCCTTAG | TCCCTGATCAATTTCTTCTTTTCT |
| YKT6 | TTGCTGACAATGAATACCCATC | TTGCTTGGAGAATTCATCTAGTAC |
